# Impact of Virtual Reality-Based Rehabilitation on Postoperative Outcomes Following Total Hip Arthroplasty: A Systematic Review and Meta-Analysis

**DOI:** 10.7759/cureus.101230

**Published:** 2026-01-10

**Authors:** Mohamed Zahed, Mahmoud Elmesalmi, Rawad Azaz, Ahmed Elkilany, Nour Elnaggar, Farouk Ahmed, Ziad El Menawy, Nada Ramadan, Manar Adel, Mahmoud M. Elhady

**Affiliations:** 1 Orthopedics, John Radcliffe Hospital, Oxford University Hospitals NHS Trust, Oxford, GBR; 2 Trauma and Orthopedics, St George's University Hospitals, London, GBR; 3 Trauma and Orthopedics, London Royal Free NHS Trust, London, GBR; 4 Trauma and Orthopedics, Worthing Hospital, University Hospitals Sussex NHS Foundation Trust, Worthing, GBR; 5 Medicine, Zagazig University, Zagazig, EGY; 6 Emergency Medicine, Queen Alexandra Hospital, Portsmouth, GBR; 7 Trauma and Orthopedics, University Hospital of Wales, Cardiff, GBR; 8 Biochemistry, Benha University, Benha, EGY; 9 Clinical Pharmacy, Tanta University, Tanta, EGY; 10 Orthopedics, Benha University, Benha, EGY

**Keywords:** immersive virtual reality, osteoarthritis hip, osteoarthritis (oa), postoperative rehabilitation, systematic review and meta analysis, total hip athroplasty

## Abstract

Hip osteoarthritis is a common cause of pain and disability, and total hip arthroplasty (THA) is an effective treatment for advanced disease. Despite surgical success, many patients experience prolonged postoperative deficits, and conventional rehabilitation does not always restore full function. Virtual reality-based rehabilitation (VR-R) has recently emerged as a potential tool to enhance recovery through increased engagement and non-pharmacological pain modulation. We aim to comprehensively evaluate the effectiveness of VR-R after THA in improving functional recovery, reducing pain and opioid use, and enhancing overall rehabilitation outcomes. We systematically searched the following databases: PubMed, Scopus, Web of Science, Cochrane Library, and Embase until November 14, 2025. The quality of the included randomized clinical trials (RCTs) was assessed using the Cochrane Risk of Bias 2 (RoB 2) tool. Statistical analysis was performed using the RevMan tool (v. 5; The Cochrane Collaboration, London, UK), with mean difference (MD) or standardized MD (SMD) for continuous data. Heterogeneity was evaluated using the I² statistic, and random-effects models were used when I² exceeded 50%. This systematic review was prospectively registered on PROSPERO (CRD420251268393). Seven studies met the inclusion criteria, comprising 397 patients. VR-R demonstrated a significant reduction in postoperative stress (SMD = -1.18; 95% confidence interval (CI): (-1.69, -0.67); p < 0.00001; I² = 0%). In contrast, no significant differences were observed between VR-R and conventional rehabilitation for pain (SMD = -0.87; p = 0.11), opioid consumption (SMD = -0.42; p = 0.40; I² = 87%), general mobility (SMD = 0.18; p = 0.32), or functional independence as measured by the Barthel index (MD = 8.00; p = 0.37). VR-R significantly reduces postoperative stress following THA but shows no significant benefits for pain, opioid consumption, mobility, or functional independence compared to standard care. Despite substantial heterogeneity across studies, VR-R represents a safe adjunctive tool for addressing psychological aspects of recovery. Standardized protocols and large-scale trials are needed to establish optimal implementation strategies.

## Introduction and background

Osteoarthritis (OA) is a chronic degenerative joint disorder characterized by the progressive deterioration of articular cartilage and associated joint structures [1]. OA is the most prevalent form of arthritis globally, affecting an estimated 350 million people, approximately 15% of the world’s population [2]. The hip is the second most frequently affected joint, following the knee [3]. Hip OA lowers quality of life due to chronic pain and stiffness, limited mobility, reduced independence, psychological burden, social withdrawal, sleep disturbance, and healthcare costs [4-6]. OA incidence increases with age, affecting more than 53% of adults aged 65 years and older [7].

However, the expectations and lifestyle standards of the elderly population have also evolved, emphasizing functional ability and well-being [8]. Given the critical role of ambulation in maintaining independence and performing daily activities, an increasing number of elderly individuals are seeking orthopedic interventions to preserve or restore their functional mobility [9]. As a result, total hip arthroplasty (THA) is now among the most commonly performed orthopedic procedures, with approximately 1.2 million THAs performed globally each year, and is highly effective for treating severe OA [10-12].

Although effective, THA is highly invasive and can result in postoperative functional limitations, with full recovery often taking up to two years [13]. These limitations include proprioceptive deficits, muscle weakness, reduced sagittal range of motion, impaired balance, increased risk of falls, persistent pain, and psychological stress [12,13]. Postoperative pain results from surgical injury, inflammation, and central sensitization, which enhances nociceptive signaling. Genetic and inflammatory factors may increase vulnerability, while psychological stressors such as anxiety, depression, and catastrophizing further amplify pain perception and contribute to chronic postsurgical pain [14]. Postoperative care typically involves analgesic therapy and rehabilitation focused on improving strength, range of motion, and overall functional capacity. Rehabilitation typically involves progressive mobility exercises, muscle strengthening, gait training, and patient education to help individuals adapt to their daily activities [15].

However, many patients still do not regain their full preoperative function, emphasizing the need for improved strategies. Emerging technologies such as biofeedback and virtual reality (VR) are increasingly employed to enhance rehabilitation [16]. This principle is grounded in the theory that complete immersion in a VR environment can reduce the brain’s capacity to perceive and interpret painful stimuli [17]. VR enables users to interact with a computer-generated environment that engages multiple sensory modalities, including visual, auditory, and haptic input. Immersion is achieved through devices such as head-mounted displays, projection systems, or sensor-based gloves that simulate realistic sensory experiences. By creating an immersive environment, VR completely isolates the user from external surroundings and fully stimulates the user’s visual, auditory, and proprioceptive senses [18,19].

The use of VR-based rehabilitation (VR-R) in orthopedic surgery, particularly after total knee arthroplasty (TKA) and THA, has increased significantly. Clinical trials demonstrated that VR shows promise as a non-pharmacological alternative to traditional pain and anxiety medications [20-22]. Despite growing interest in VR applications across orthopedic surgery, there remains limited evidence specifically examining its role in THA. While VR has shown promise in preoperative planning and surgical training for various procedures, comprehensive studies evaluating its effectiveness are lacking [23]. This study aims to evaluate the effectiveness of VR-R following THA, specifically determining the impact of VR-R interventions on postoperative functional recovery, pain reduction, opioid use, and overall rehabilitation outcomes compared with conventional rehabilitation methods.

## Review

Methods

This systematic review and meta-analysis was performed following the Cochrane Handbook for Systematic Reviews of Interventions and adhered to the Preferred Reporting Items for Systematic Reviews and Meta-Analyses (PRISMA) guidelines [24,25]. This systematic review was prospectively registered on PROSPERO (CRD420251268393).

Search Strategy and Data Collection

We systematically searched the following databases: PubMed, Scopus, Web of Science, Cochrane Library, and Embase until November 14, 2025. The following words were used in search strategy: (Hip) AND (Arthroplasty OR Replacement OR Implantation OR Hemiarthroplasty OR Reconstruction OR Prosthesis OR Endoprosthesis) AND ("virtual reality" OR VR OR "artificial reality" OR simulation OR "Simulated reality" OR "Computer-simulated environment" OR "Virtual simulation" OR "artificial environment" OR "computerized simulation" OR "simulated reality" OR "augmented reality" OR "virtual environment" OR "computer simulation" OR "simulated 3D environment" OR "virtual world" OR "extended reality" OR virch OR "mixed reality" OR XR OR "simulated experience") AND (random* OR RCT OR "clinical trial"). A detailed search strategy is available in the Appendix.

Selection and Eligibility Criteria

We included only randomized clinical trials (RCTs) that met the following criteria: (1) population: patients who underwent hip arthroplasty, (2) intervention: the use of VR, (3) comparator group: standard care, traditional training or rehabilitation methods, or other non-virtual/computerized approaches, and (4) outcomes: pain score, general mobility, Barthel index (0-100), stress, and opioid consumption. Studies were excluded if they met any of the following criteria: (1) non-randomized study designs including observational studies, case reports, case series, and letters to the editor; (2) studies involving populations other than hip arthroplasty patients (e.g., knee arthroplasty alone, spine surgery, or other orthopedic procedures); (3) interventions not primarily based on VR technology; (4) absence of a comparator group or use of another VR modality as the sole comparator; (5) studies not reporting at least one of the prespecified outcomes; (6) conference abstracts without full-text availability; (7) duplicate publications or secondary analyses of previously included trials; and (8) non-English-language publications without available translations. Two different authors performed the screening, with the third author resolving any disagreement between them.

Data Extraction

Data were extracted using predefined Excel forms (Microsoft Corp., Redmond, WA, US) and organized into two primary categories: (1) study summary, including study ID, details of the intervention and comparator, timing of VR initiation, and frequency of VR use, and (2) baseline participant characteristics, including study ID, sample size per group, mean age ± standard deviation (SD), sex distribution (female, n (%)), body mass index (BMI), and baseline hip function scores. Two independent reviewers performed the data extraction.

Quality Assessment

The methodological quality of the included RCTs was assessed using the Cochrane Risk of Bias 2 (RoB 2) tool [26]. This tool evaluates five key domains: (1) the randomization process, (2) deviations from intended interventions, (3) missing outcome data, (4) outcome measurement, and (5) the selection of reported results. Based on these domains, each study was classified as having low, some concerns, or high risk of bias. Two independent reviewers did the quality assessment, with a third reviewer resolving any disagreements.

Statistical Analysis

We used the RevMan tool (v. 5; The Cochrane Collaboration, London, UK) to perform the statistical analysis. The result was considered significant when p < 0.05. The mean difference (MD) with its 95% confidence interval (CI) was calculated for continuous outcomes measured using the same scale. When studies assessed the same outcome using different measurement tools, the standardized MD (SMD) was applied instead. Heterogeneity across the included studies was evaluated using the I² statistic and the chi-squared test. Statistical heterogeneity was considered present when the chi-squared test produced a p-value < 0.1 and the I² value exceeded 50%. A fixed-effect model was applied when no substantial heterogeneity was detected, whereas a random-effects model was used when significant heterogeneity was identified. Statistical heterogeneity was assessed using the I² statistic and interpreted according to the Cochrane Handbook guidelines: 0%-40% may not be important, 30%-60% represents moderate heterogeneity, 50%-90% indicates substantial heterogeneity, and 75%-100% reflects considerable heterogeneity.

Sensitivity analyses were planned, including exclusion of studies at high risk of bias and assessment of publication bias using funnel plots when at least 10 studies were available. Meta-regression analyses were planned to explore potential sources of heterogeneity; however, these analyses were not feasible due to the limited number of included studies. Assessment of the certainty of evidence using the GRADE approach was considered; however, formal grading was limited by the small number of studies and substantial heterogeneity.

Outcome Measures

The level of functional independence in daily living activities was evaluated using the Barthel index. It is a standardized tool comprising 10 domains that measure basic daily activities such as feeding, bathing, dressing, toileting, continence, transfers, and walking. Each domain score ranges from 0 to 10, with a maximum of 100. The higher scores indicate greater functional independence, while lower scores indicate functional limitations [27].

Postoperative pain was measured using validated tools, including the Numeric Rating Scale (NRS), where pain intensity is rated from 0 (no pain) to 10 (worst possible pain), and the Visual Analogue Scale (VAS), a 10 cm line anchored at 0 (no pain) and 10 (worst imaginable pain), with lower values indicating better pain control [28]. In some cases, pain was evaluated using the WOMAC pain subscale, comprising five items scored from 0 to 20, with higher scores indicating greater pain severity [29].

Mobility outcomes were assessed using several standardized measures. The Tinetti Short Scale, a simplified version of the Tinetti Performance-Oriented Mobility Assessment, evaluates gait and balance through tasks such as sit-to-stand transitions, standing for 5 s, walking 3 m, performing a 180° turn, and sitting down again. Each task is scored on a 3-point Likert scale, producing a total score from 0 to 15, with higher scores indicating better mobility [30]. Mobility was also measured using the “6 Clicks” Basic Mobility Short Form, which assesses six essential mobility tasks and generates a score ranging from 6 to 24, with higher scores reflecting better functional mobility [31].

Psychological stress was evaluated using validated instruments. The Perception of Stress Questionnaire (PSQ) measures stress across three domains (emotional tension, external stressors, and intrapsychic stress) and provides a total score between 21 and 105, with higher scores indicating greater stress levels [32]. Additionally, the Perceived Stress Scale (PSS-10) was used to assess perceived stress over the past month through 10 broadly phrased items, making it suitable for diverse populations [33].

Results

Literature Search and Study Selection

**Figure 1:**
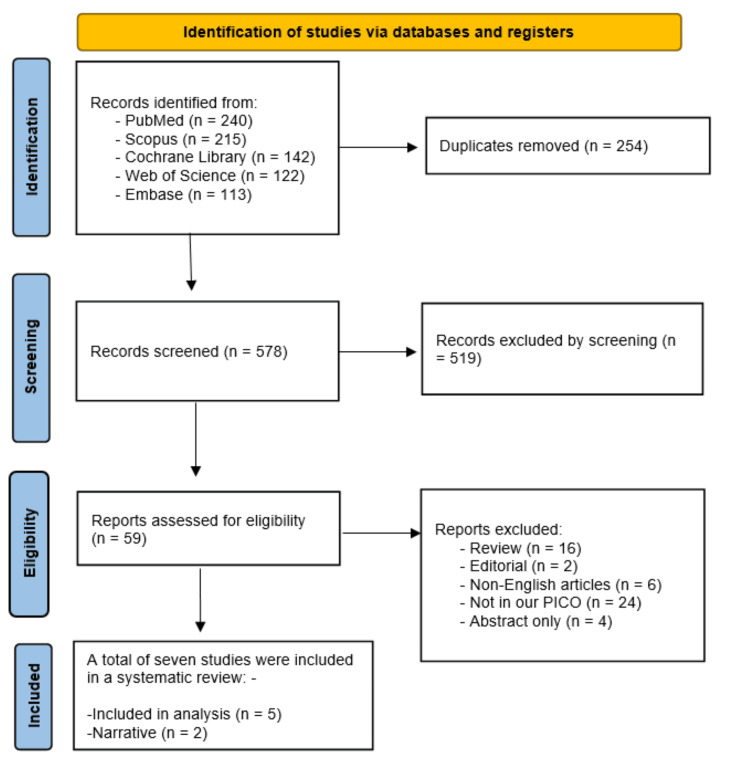
PRISMA flow diagram PRISMA: Preferred Reporting Items for Systematic Reviews and Meta-Analyses; PICO: population, intervention, comparator, and outcome

Using our search strategy, we identified 578 records after removing 254 duplicates. After screening titles and abstracts, we selected 59 articles for full-text review. Of these, seven studies met the inclusion criteria and were included [34-40]. Two studies were included narratively, while five were included in the analysis (Figure 1).

Study Summary Characteristics

All the included studies used VR similarly, providing immersive, interactive experiences rather than passive content. Every study aimed to achieve clinical benefits, whether psychological, functional, or pain-related, and measured outcomes systematically to evaluate effectiveness. The studies differed substantially in three key areas. First, timing varied widely: some used VR preoperatively (Moharam 2025) [38], others started immediately postoperatively on day one or two (Fascio 2022, Araujo-Duran 2024, and Lehrl 2012) [34-36], while some implemented it only during the rehabilitation phase (Mazurek 2023, Zavala-Gonzalez 2025) [39,40]. The intervention characteristics ranged from high-end headsets to Nintendo gaming consoles; content included therapeutic gardens, cognitive games, balance exercises, and surgical simulations; and the duration ranged from a single 15-minute session to a daily 30-minute program over six weeks (Table 1).

**Table 1:** Summary of the included studies References [34-40] VR: virtual reality; THA: total hip arthroplasty; VR-R: virtual reality rehabilitation; HTC VIVE: VR headset brand; DS: Nintendo DS gaming device; NCT: National Clinical Trial registration; ACTRN: Australian New Zealand Clinical Trials Registry; 2D: two-dimensional; 3D: three-dimensional

Study ID	Registration	VR intervention details	Comparator details	When VR performed (pre-/post-op)	Why VR performed	Duration/frequency
Araujo-Duran 2024 [35]	NCT04416555	Device: Pico G2 4K headset with AppliedVR software. Content: 3D immersive VR with guided breathing exercises, games, mindfulness, and relaxation programs	Device: identical Pico G2 4K headset. Content: 2D nature short films (forest wildlife) with neutral music, non-interactive, presented on a virtual screen	Postoperative: starting postoperative day 1 through day 2 or discharge	Primary aim: reduce acute postoperative pain and opioid requirements. Mechanism: relaxation and distraction to provide analgesia	Three sessions/day targeted ≥ 1 h between sessions used during physical therapy or ambulation
Fascio 2022 [34]	NCT04221425	Device: VRRS (Virtual Reality Rehabilitation System) by Khymeia Group Components: tablet with wearable sensors. Content: interactive virtual exercise program for hip rehabilitation after THA	Method: illustrated exercise booklet. Content: same exercises as the VR group, but self-directed. Instruction: as much as you can (AMAYC) approach	Postoperative: starting postoperative day 2. Through day 15 ± 2 or discharge	Primary aim: improve functional outcomes and hip disability scores. Additional goals: enable remote monitoring, increase patient compliance, modernize rehabilitation delivery via telerehabilitation	Daily 30+ min sessions VR: 40 reps/5 min (week 1), 80 reps/10 min (week 2)
Lehrl 2012 [36]	NA	Device: Nintendo DS with Dr. Kawashima's Brain Training game. Content: mental task challenge information processing speed and memory span. Tasks: quick arithmetic, number sequencing, tracking people entering/leaving the house. Goal: use full working memory capacity	Method: no gaming intervention. Activity: routine hospital care only	Postoperative: starting postoperative day 2. Through day 12 ± 1	Primary aim: test if mental activation supports physical rehabilitation. Mechanism: mental activation may reduce pain perception, increase participation in physical therapy, and improve excitability and motivation	Daily 30+ min sessions under the supervision of the study nurse. First session on admission day (pre-op) for training
Mazurek 2023 [39]	NCT06002139	Device: VR Tier One system (HTC VIVE goggles + controllers). Content: immersive therapeutic garden environment that progressively evolves across sessions. Midway through each session, participants color a mandala using controllers. Goal: reduce anxiety, depression, and stress in elderly patients post-THA through attention diversion, relaxation, and recognition of psychological strengths	Standard rehabilitation alone: 2 h kinesiotherapy (120 min gait training), 30 min ergotherapy, physical therapy (laser, magnetic, electrotherapy)	Postoperative rehabilitation program	Primary goal: reduce anxiety, depression, and stress post-THA. Mechanism: immersive relaxation environment, attention diversion, psychological strengthening through metaphorical communication	Total: 4 weeks. VR sessions: 8 sessions (20 min, 2×/week). Standard rehab: daily
Moharam 2025 [38]	NCT06088069	Device: VR headset with audio headset. Content: serene natural environment with soft music, providing complete isolation from the actual surgical environment. Goal: reduce perioperative anxiety, stress, and pain through immersive distraction and relaxation during the surgical experience	Standard physiotherapy: spinal anesthesia without intrathecal adjuvants, routine postoperative care	Preoperative: 15 min before surgery. Intraoperative: during THA surgery. Follow-up: outcomes assessed immediately post-op	Primary goal: reduce perioperative anxiety, stress, and pain. Mechanism: immersion in a calming environment to isolate from surgical reality, provide distraction	15 min (before spinal anesthesia) and continuous during the entire THA surgery (approximately 90-120 min based on typical THA duration)
Szczepańska-Gieracha 2025 [37]	NCT06506760	Device: VRTierOne medical device (HTC VIVE goggles + HTC VIVE controller tracking dominant wrist). Content: patient enters through a virtual gate into a dynamically developing garden that becomes increasingly vibrant with each session. Background music progresses from relaxation to uplifting. Goal: improve psychological well-being and functional outcomes in elderly women post-THA through calming virtual immersion and psychological strengthening	Standard rehabilitation: 2 h kinesiotherapy (gait training), 30 min ergotherapy, physical therapy (laser, cryotherapy, magnetic, electrotherapy)	Postoperative, all participants were service users attending the postoperative rehabilitation service after THA	Primary goal: improve psychological well-being and functional status post-THA. Mechanism: interactive immersive environment simulating real-world experiences safely, promoting efficient recovery through controlled sensorimotor stimuli	Total: 4 weeks. VR sessions: 8 sessions (20 min, 2×/week). Standard rehab: daily
Zavala-Gonzalez 2025 [40]	ACTRN12618001252202	Device: Nintendo Wii with Wii Balance Board and controllers. Content: two balance-focused games: tight rope walk: advance along the tight rope by shifting the center of gravity left/right within 45 s. Balance bubble: navigate a soap bubble along the river by shifting the center of gravity across all planes within 1 min. Goal: improve lower limb function, balance, and postural control through real-time center-of-pressure biofeedback and sensorimotor training	Conventional physiotherapy: hot compress (10 min), cycle ergometer (10 min), quadriceps strengthening, hip abduction, glute bridge, lateral march, walking training	Postoperative	Primary goal: improve lower limb physical function, balance, and pain management. Mechanism: interactive feedback for functional training, real-time movement correction, enhanced motor control efficiency through different sensorimotor mechanisms	Total: 6 weeks. VR sessions: 12 sessions (15 min, 2×/week). Conventional therapy: each session

Baseline Characteristics

Baseline characteristics were clinically comparable across studies. Among the included studies, sample sizes ranged from 12 to 54 participants, with most enrolling 16-37 patients per group. Mean age at baseline varied from 66 ± 10 to 72.17 ± 7.40 years. The proportion of female participants ranged between 37.5% and 67% across studies. BMI ranged from 25.76 ± 4.17 to 31.5 ± 7 kg/m². Baseline hip function was assessed using different validated instruments, including the Harris Hip Score, HOOS-JR, WOMAC function subscale, and the Barthel index, with values indicating moderate functional limitation at entry (Table 2).

**Table 2:** Baseline characteristics References [34-40] BMI: body mass index; HOOS JR: Hip disability and Osteoarthritis Outcome Score for Joint Replacement; Harris Hip: Harris Hip Score; WOMAC: Western Ontario and McMaster Universities Arthritis Index; SD: standard deviation

Study ID	Sample size	Age (years) mean ± SD	Sex (% female)	BMI mean ± SD	Hip function baseline	Diabetes (%)
Araujo-Duran 2024 [35]	52	66 ± 10	27 (52%)	32 ± 6	Harris Hip: 62.23 ± 15.54	10 (19.2%)
	54	63 ± 9	25 (46%)	31.5 ± 7	Harris Hip: 62.71 ± 11.74	12 (22.2%)
Fascio 2022 [34]	21	66.1 ± 9.3	12 (57.1%)	NA	HOOS JR Pre-op: 38.9 ± 14.3	5 (22.2%)
	22	68.9 ± 13.9	12 (56.5%)	NA	HOOS JR Pre-op: 32.9 ± 9.9	4 (19.2%)
Lehrl 2012 [36]	16	66.1 ± 9.3	6 (37.5%)	NA	Harris Hip: 39	3 (19.2%)
	16	68.9 ± 13.9	6 (37.5%)	NA	Harris Hip: 33	4 (22.2%)
Mazurek 2023 [39]	34	71.91 ± 10.18	20 (60%)	28.69 ± 4.38	WOMAC function 52.05 ± 4.07	NA
	34	72.17 ± 7.40	21 (62.16%)	29.07 ± 5.73	WOMAC function 52.57 ± 4.93	NA
Moharam 2025 [38]	25	69.71 ± 6.82	15 (61.76%)	28.46 ± 5.25	WOMAC function 52.05 ± 4.07	7 (28%)
	25	69.47 ± 5.52	15 (61.76%)	28.62 ± 5.05	WOMAC function 53.46 ± 4.27	5 (20.83%)
Szczepańska-Gieracha 2025 [37]	12	71.91 ± 10.18	7 (60%)	28.69 ± 4.38	Barthel index 55.91 ± 19.34	NA
	12	72.17 ± 7.40	7 (60%)	29.07 ± 5.73	Barthel index 56.18 ± 17.41	NA
Zavala-Gonzalez 2025 [40]	37	67.68 ± 8.37	25 (67.57%)	25.76 ± 4.17	WOMAC function 52.57 ± 4.93	NA
	37	71.18 ± 9.16	22 (59.46%)	27.22 ± 4.79	WOMAC function 53.46 ± 4.27	NA

Quality of the Included Studies

**Figure 2:**
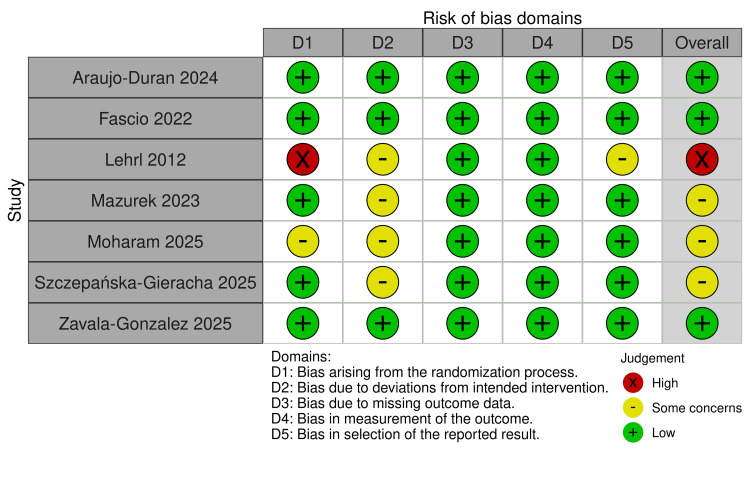
Risk of Bias 2 References [34-40]

Three studies were rated as having a low risk of bias, whereas three others were judged to have some concerns. In these studies, the D2 domain was not rated as low risk because adherence to the assigned interventions was not clearly reported, and the handling of any deviations from the intended intervention was insufficiently described. One study has a high risk of bias related mainly to the randomization process (D1) (Figure 2).

Primary outcomes

Pain Improvement

**Figure 3:**
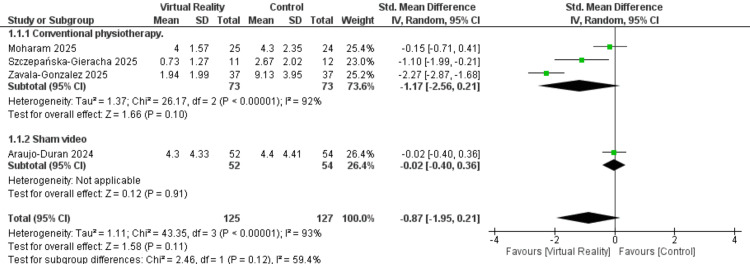
Pain score References [35,37,38,40] SD: standard deviation; CI: confidence interval

Pain outcomes were reported in four studies, including a total of 252 patients. The overall pooled analysis demonstrated no significant difference between the VR-R and control groups (SMD = -0.87; 95% CI: (-1.95, 0.21); p = 0.11), with substantial heterogeneity observed (I² = 93%, p < 0.0001). A prespecified subgroup analysis was conducted, comparing (1) VR-R combined with conventional physiotherapy versus conventional physiotherapy alone and (2) sham video versus control. Neither subgroup showed a statistically significant effect, with results of SMD = -1.17 (95% CI: (-2.56, 0.21); p = 0.10; I² = 92%, p < 0.00001) for subgroup 1 and SMD = −0.02 (95% CI: (-0.40, 0.36); p = 0.91; I² = 0%) for subgroup 2. The test for subgroup differences was not statistically significant (p = 0.12), indicating that the observed variation in effect estimates between subgroups may be due to chance rather than a true differential effect (Figure 3).

Opioid Consumption

**Figure 4:**
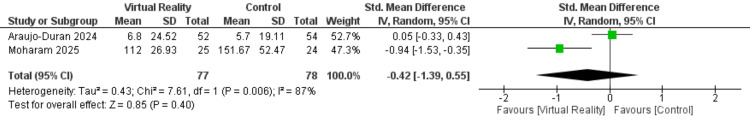
Opioid consumption (mg) References [35,38] SD: standard deviation; CI: confidence interval

Two of the included studies, comprising 155 participants, reported opioid consumption. The results showed no statistical significance between the VR-R and control groups, with an SMD of -0.42 (95% CI: (-1.39, 0.55); p = 0.40). Substantial heterogeneity was observed among the included studies (I² = 87%, p = 0.006) (Figure 4).

Decrease in Stress

**Figure 5:**
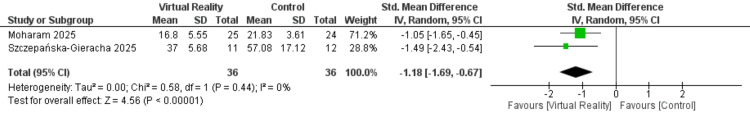
Decrease in stress References [37,38] SD: standard deviation; CI: confidence interval

Two studies involving 72 patients assessed postoperative stress-related outcomes. The pooled analysis showed a significant advantage in favor of the VR-R group compared with the control group (SMD = -1.18; 95% CI: (-1.69, -0.67); p < 0.00001), with no heterogeneity detected between studies (I² = 0%, p = 0.44) (Figure 5).

General Mobility

**Figure 6:**
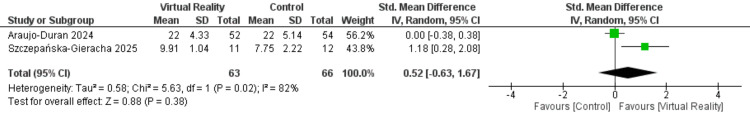
General mobility References [35,37] SD: standard deviation; CI: confidence interval

Two studies involving 129 participants assessed general mobility outcomes. The pooled analysis revealed no significant difference between the VR-R and control groups (SMD = 0.18; 95% CI: (-0.17, 0.53); p = 0.32). Substantial heterogeneity was observed across the studies (I² = 82%, p = 0.02) (Figure 6).

Barthel Index (0-100)

**Figure 7:**
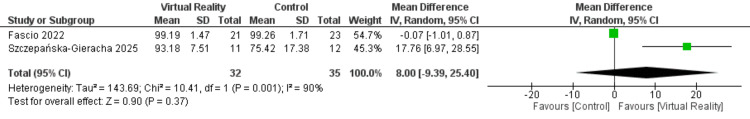
Barthel index (0-100) References [34,37] SD: standard deviation; CI: confidence interval

Two studies, comprising 67 participants in total, evaluated functional independence using the Barthel index. The pooled analysis showed no significant difference between the VR-R and control groups (MD = 8.00; 95% CI: (-9.39, 25.40); p = 0.37). Substantial heterogeneity was detected (I² = 90%, p = 0.001) (Figure 7).

Qualitative Studies

Lehrl et al. [36] is an RCT that was conducted in 32 patients undergoing THA, all performed using a standardized surgical approach. Analgesic medications with strong central effects were excluded, and all participants received the same postoperative pain management regimen. The intervention consisted of a cognitive video game, Dr. Kawashima’s Brain Training: How Old Is Your Brain? (Nintendo, Kyoto, Japan). Patients in the intervention group demonstrated significantly greater postoperative functional gains compared with controls. At discharge, they achieved higher Harris Hip Scores (76.0 vs. 56.5; p = 0.001) and Merle Aubigné Scores (16.0 vs. 13.5; p = 0.014). Both groups showed improvement following surgery, but the magnitude of recovery was markedly greater in the mental activation group.

Mazurek et al. [39] is an RCT that included 68 participants who underwent hip or knee joint arthroplasty. The interventional group (VR-R group) included 34 patients. The experimental group showed notable enhancements in both psychological and functional areas compared to the control group. The VR-R intervention produced significant improvements across psychological outcomes. Compared with the control group, the VR-R group showed greater reductions in anxiety and depression (HADS-Total: -8.66, p < 0.001; HADS-A: -4.37, p < 0.001; HADS-D: -4.27, p < 0.001). Perceived stress also declined substantially only in the VR-R group (PSS-10: -3.30, p < 0.001; PSQ: −23.91, p < 0.001). Self-efficacy increased significantly following VR-R therapy (+10.4 points on the General Self-Efficacy Scale (GSES)), whereas controls showed no improvement. Also, pain (VAS) decreased in both groups, but the VR-R group experienced a significantly greater reduction (-2.63; p < 0.001). Functional outcomes also favored the VR-R group. With significantly greater improvements in the Barthel index, the VR group showed a markedly larger gain (between-group difference 21.18; p < 0.001). Balance and gait performance, measured by the Tinetti Short Scale, also improved more in the VR-R group (3.41; p < 0.001). Mobility outcomes assessed using the Rivermead Mobility Assessment demonstrated superior improvement in the VR-R group (2.04; p < 0.001). Similarly, physical performance, as measured by the Short Physical Performance Battery (SPPB), showed a substantial advantage for the VR-R group (3.79; p < 0.001).

Discussion

This systematic review and meta-analysis evaluated the effectiveness of VR-R following THA. The pooled analysis revealed that VR-R significantly reduced postoperative stress compared with conventional rehabilitation. In contrast, VR-R did not demonstrate significant benefits over standard care for pain reduction, opioid consumption, general mobility, or functional independence measured by the Barthel index. Findings from narratively synthesized studies supported these trends. By reporting potential improvements in functional outcomes in the VR-R group, showing trends toward better psychological and functional measures, most outcomes did not reach statistical significance, and results should be interpreted with caution due to substantial heterogeneity.

A recent systematic review and meta-analysis by García-Sánchez et al. examined the effectiveness of VR-based therapy following THA, including five RCTs with 287 patients. Their meta-analysis demonstrated that non-immersive VR-R combined with physiotherapy significantly reduced hip disability (SMD = -0.46; p = 0.018) and improved hip function (SMD = 0.6; p = 0.002) compared to physiotherapy alone. Qualitative synthesis of that review suggested that VR-based cognitive exercises could enhance disability outcomes, physical function, and subjective perceptions of improvement [41].

Our findings on stress reduction align with evidence from Gumaa and Rehan Youssef's systematic review, which highlighted VR's potential in orthopedic rehabilitation. The authors noted that VR-R enables personalized treatment, increases patient motivation and compliance, requires minimal supervision, and is suitable for home-based use. These features are particularly relevant to post-THA rehabilitation, where patient engagement significantly influences recovery. The stress reduction observed in our meta-analysis may be attributed to VR's immersive environment that modulates pain perception through central neural mechanisms [42].

VR-R can be classified as immersive or non-immersive based on the level of sensory engagement provided. Non-immersive systems present virtual content on a standard screen with interaction limited to handheld controllers or conventional input devices. In contrast, immersive VR-R systems utilizing head-mounted displays envelop the user in a three-dimensional environment that responds dynamically to head and body movements. Immersive systems facilitate greater sensory-motor integration and a heightened sense of presence, both of which have been associated with superior therapeutic effects. This deeper engagement may enhance attentional diversion from pain and promote greater emotional regulation compared to non-immersive alternatives [43].

The psychological benefits of VR-R may extend beyond stress reduction to include enhanced motivation and treatment adherence. Previous research has demonstrated that VR-R increases patient motivation and engagement with therapy, which may translate into improved functional recovery outcomes. Motivation represents a critical psychological determinant in rehabilitation that can substantially influence patient recovery trajectories [44]. The gamified nature of VR-R interventions reduces the monotony associated with conventional rehabilitation protocols, thereby promoting sustained participation and adherence to prescribed exercise regimens. This increased engagement may partially explain the functional improvements observed in our narratively synthesized studies, where patients receiving VR-based interventions demonstrated superior outcomes compared to those receiving standard care alone [45].

Postoperative rehabilitation following THA traditionally relies on conventional physical therapy, including supervised exercises, manual therapy, and progressive mobilization protocols. However, technology-based rehabilitation interventions, including telerehabilitation and VR-R, are increasingly being explored across various populations to enhance functional recovery outcomes [46]. Similarly, the integration of emerging technologies in joint arthroplasty extends to surgical approaches, with robotic-assisted techniques demonstrating improved precision and alignment accuracy in TKA [47].

The included studies employed various validated tools to assess pain outcomes. Pain intensity was measured using the Numeric Rating Scale, VAS, and WOMAC pain subscale. While these instruments differ in format and scoring, all are well-established measures of pain severity with demonstrated validity in postoperative populations. The use of different scales necessitated the application of SMDs in our pooled analysis to allow for meaningful comparisons across studies.

Mobility and stress outcomes were similarly assessed using heterogeneous instruments. Mobility was evaluated using the Tinetti Short Scale and the "6 Clicks" Basic Mobility Short Form, both of which assess functional mobility but differ in their scoring ranges and specific components evaluated. Stress was measured using the PSQ and the PSS, which assess psychological stress through different domains and timeframes. This variability in outcome measurement tools across studies may have contributed to the observed heterogeneity and should be taken into account when interpreting the pooled results.

Our study has several strengths, as it consisted only of RCTs, which ensured the high-quality evidence. The sample size is sufficient for developing reliable evidence. This study addresses a vital evidence gap regarding VR-R, specifically following THA. On the other hand, the study had several limitations, including a small number of included studies, which limited generalizability. Substantial heterogeneity was observed across most outcomes, likely due to variability in VR-R type, timing, duration, and outcome measures. Studies used a range of VR modalities, from immersive head-mounted displays to non-immersive gaming systems. Blinding was not possible due to the nature of VR-R interventions. Most studies had short follow-up periods, and publication bias cannot be excluded. Additionally, one of the included studies contained both TKA and THA, so we included it narratively only.

Large-scale, multicenter RCTs with standardized VR-R protocols and consistent outcome measures are needed. Long-term follow-up assessments should be conducted to evaluate sustained benefits. Comparative studies examining immersive versus non-immersive systems would clarify which modalities are most effective.

## Conclusions

Our study suggests that VR-R interventions may offer potential benefits in reducing postoperative stress compared to conventional rehabilitation approaches; however, these findings should be interpreted with caution, given the limited number of included studies, small sample sizes, and substantial heterogeneity. VR-R did not show statistically significant advantages over standard care for pain reduction, opioid consumption, general mobility, or functional independence. The variability in VR-R modalities, timing of implementation, intervention duration, and outcome measurement tools across studies limits the generalizability of our results. Future research should focus on large-scale, multicenter randomized controlled trials with standardized VR-R protocols and consistent outcome measures to establish optimal implementation strategies. Additionally, VR-R may represent a safe and potentially beneficial adjunctive tool for addressing psychological aspects of post-THA recovery, though further high-quality evidence is needed.

## Appendices

**Table 3:** Detailed search strategy

Database	Number of results	Search strategy
PubMed	240	(Hip) AND (Arthroplasty OR Replacement OR Implantation OR Hemiarthroplasty OR Reconstruction OR Prosthesis OR Endoprosthesis) AND ("virtual reality" OR VR OR "artificial reality" OR simulation OR "Simulated reality" OR "Computer-simulated environment" OR "Virtual simulation" OR "artificial environment" OR "computerized simulation" OR "simulated reality" OR "augmented reality" OR "virtual environment" OR "computer simulation" OR "simulated 3D environment" OR "virtual world" OR "extended reality" OR virch OR "mixed reality" OR XR OR "simulated experience") AND (random* OR RCT OR "clinical trial")
Scopus	215	TITLE-ABS-KEY ( ( Hip ) AND ( Arthroplasty OR Replacement OR Implantation OR Hemiarthroplasty OR Reconstruction OR Prosthesis OR Endoprosthesis ) AND ( "virtual reality" OR VR OR "artificial reality" OR simulation OR "Simulated reality" OR "Computer-simulated environment" OR "Virtual simulation" OR "artificial environment" OR "computerized simulation" OR "simulated reality" OR "augmented reality" OR "virtual environment" OR "computer simulation" OR "simulated 3D environment" OR "virtual world" OR "extended reality" OR virch OR "mixed reality" OR XR OR "simulated experience" ) AND ( random* OR RCT OR "clinical trial" ) )
Web of Science	122	TS=((Hip) AND (Arthroplasty OR Replacement OR Implantation OR Hemiarthroplasty OR Reconstruction OR Prosthesis OR Endoprosthesis) AND ("virtual reality" OR VR OR "artificial reality" OR simulation OR "Simulated reality" OR "Computer-simulated environment" OR "Virtual simulation" OR "artificial environment" OR "computerized simulation" OR "simulated reality" OR "augmented reality" OR "virtual environment" OR "computer simulation" OR "simulated 3D environment" OR "virtual world" OR "extended reality" OR virch OR "mixed reality" OR XR OR "simulated experience") AND (random* OR RCT OR "clinical trial"))
Cochrane Library	142	((Hip) AND (Arthroplasty OR Replacement OR Implantation OR Hemiarthroplasty OR Reconstruction OR Prosthesis OR Endoprosthesis) AND ("virtual reality" OR VR OR "artificial reality" OR simulation OR "Simulated reality" OR "Computer-simulated environment" OR "Virtual simulation" OR "artificial environment" OR "computerized simulation" OR "simulated reality" OR "augmented reality" OR "virtual environment" OR "computer simulation" OR "simulated 3D environment" OR "virtual world" OR "extended reality" OR virch OR "mixed reality" OR XR OR "simulated experience") AND (random* OR RCT OR "clinical trial")):ti,ab,kw
Embase	113	hip:ab,ti AND (arthroplasty:ab,ti OR replacement:ab,ti OR implantation:ab,ti OR hemiarthroplasty:ab,ti OR reconstruction:ab,ti OR prosthesis:ab,ti OR endoprosthesis:ab,ti) AND ('virtual reality':ab,ti OR vr:ab,ti OR 'artificial reality':ab,ti OR simulation:ab,ti OR 'computer-simulated environment':ab,ti OR 'virtual simulation':ab,ti OR 'artificial environment':ab,ti OR 'computerized simulation':ab,ti OR 'simulated reality':ab,ti OR 'augmented reality':ab,ti OR 'virtual environment':ab,ti OR 'computer simulation':ab,ti OR 'simulated 3d environment':ab,ti OR 'virtual world':ab,ti OR 'extended reality':ab,ti OR virch:ab,ti OR 'mixed reality':ab,ti OR xr:ab,ti OR 'simulated experience':ab,ti) AND (random*:ab,ti OR rct:ab,ti OR 'clinical trial':ab,ti)
